# Determinants and Interactions of Oral Bacterial and Fungal Microbiota in Healthy Chinese Adults

**DOI:** 10.1128/spectrum.02410-21

**Published:** 2022-02-02

**Authors:** Man Kit Cheung, Jason Y. K. Chan, Martin C. S. Wong, Po Yee Wong, Pu Lei, Liuyang Cai, Linlin Lan, Wendy C. S. Ho, Apple C. M. Yeung, Paul K. S. Chan, Zigui Chen

**Affiliations:** a Department of Microbiology, Faculty of Medicine, The Chinese University of Hong Kong, Hong Kong Special Administrative Region, China; b Department of Otorhinolaryngology, Head and Neck Surgery, Faculty of Medicine, The Chinese University of Hong Kong, Hong Kong Special Administrative Region, China; c Jockey Club School of Public Health and Primary Care, The Chinese University of Hong Kong, Hong Kong Special Administrative Region, China; d Stanley Ho Centre for Emerging Infectious Diseases, Faculty of Medicine, The Chinese University of Hong Kong, Hong Kong Special Administrative Region, China; Quest Diagnostics Nichols Institute

**Keywords:** oral microbiome, bacterial microbiota, mycobiome, bacterial–fungal interaction, keystone species

## Abstract

Numerous studies have examined the composition of and factors shaping the oral bacterial microbiota in healthy adults; however, similar studies on the less dominant yet ecologically and clinically important fungal microbiota are scarce. In this study, we characterized simultaneously the oral bacterial and fungal microbiomes in a large cohort of systemically healthy Chinese adults by sequencing the bacterial 16S rRNA gene and fungal internal transcribed spacer. We showed that different factors shaped the oral bacterial and fungal microbiomes in healthy adults. Sex and age were associated with the alpha diversity of the healthy oral bacterial microbiome but not that of the fungal microbiome. Age was also a major factor affecting the beta diversity of the oral bacterial microbiome; however, it only exerted a small effect on the oral fungal microbiome when compared with other variables. After controlling for age and sex, the bacterial microbiota structure was most affected by marital status, recent oral conditions and oral hygiene-related factors, whereas the fungal microbiota structure was most affected by education level, fruits and vegetables, and bleeding gums. Bacterial–fungal interactions were limited in the healthy oral microbiota, with the strongest association existing between Pseudomonas sp. and *Rhodotorula dairenensis*. Several bacterial amplicon sequence variants (ASVs) belonging to Veillonella atypica and the genera *Leptotrichia*, Streptococcus and *Prevotella_7* and fungal ASVs belonging to Candida albicans and the genus *Blumeria* were revealed as putative pivotal members of the healthy oral microbiota. Overall, our study has facilitated understanding of the determining factors and cross-kingdom interactions of the healthy human oral microbiome.

**IMPORTANCE** Numerous studies have examined the bacterial community residing in our oral cavity; however, information on the less dominant yet ecologically and clinically important fungal members is limited. In this study, we characterized simultaneously the oral bacterial and fungal microbial communities in a large cohort of healthy Chinese adults, examined their associations with an array of host factors, and explored potential interactions between the two microbial groups. We showed that different factors shape the diversity and structure of the oral bacterial and fungal microbial communities in healthy adults, with, for instance, sex and age only associated with the diversity of the bacterial community but not that of the fungal community. Besides, we found that bacterial–fungal interactions are limited in the healthy oral cavity. Overall, our study has facilitated understanding of the determining factors and bacterial–fungal interactions of the healthy human oral microbial community.

## INTRODUCTION

The human oral cavity is among the most diverse microbial ecosystems in the human body, together with the gut (1), harboring over 600 bacterial species and 100 fungal species (2, 3). Microbes residing in the human oral cavity, collectively known as the oral microbiota, play crucial roles in health and disease (4). Numerous efforts have been made to characterize the composition and determinants of the normal oral bacterial microbiome in healthy adults (5–7). However, the oral fungal microbiome, also known as mycobiome, in healthy adults has received much less attention (3, 8), albeit their ecological and clinical significance (9). As a result, factors shaping the healthy oral mycobiome remain largely elusive to date. Besides, potential interactions between the two kingdoms in the oral microbiome of healthy individuals remain largely unexplored. A comprehensive understanding of the influence of the human oral microbiota on health and disease requires a holistic view of both intra- and cross-kingdom interactions among members of the oral microbiota (10). In fact, based on the analysis of ecological association networks, it has been illustrated that characterization of the relationships among members of a microbial ecosystem is biased unless members from other kingdom(s) are also included (11).

In this study, we characterized simultaneously for the first time the oral bacterial and fungal microbiomes in a large cohort of systemically healthy Chinese adults by using high-throughput next-generation sequencing. We examined the associations between 24 metadata variables related to sociodemographic characteristics, lifestyle, diet, oral hygiene, oral health, and oral intimate behavior and the oral bacterial and fungal microbiomes. We further elucidated bacterial–fungal interactions and identified potential keystone taxa of the healthy oral microbiome by constructing cross-kingdom association networks.

## RESULTS

**FIG 1:**
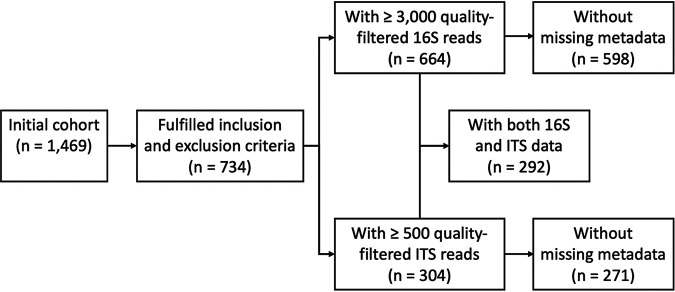
Flow chart of sample selection for the current study. Quality-filtered 16S rRNA and internal transcribed spacer (ITS) sequence data sets were used in analysis of cohort characteristics, rarefaction curves, and taxonomic barplots, whereas subsets with complete metadata were used in diversity analysis, effect size analysis, and differential abundance analysis. Samples with both 16S and ITS sequence data were used in network analysis.

After sample selection and quality filtering, 16S rRNA gene sequence data from 664 healthy individuals were included in bacterial microbiome analysis and ITS sequence data from 304 healthy individuals were included in mycobiome analysis (Fig. 1). Both populations had a sex ratio of 1:1 and a mean age of around 45 yrs (range: 18–65 yrs) (Table S1 in the supplemental material). Detailed characteristics of the study cohort are given in Table S1. Rarefaction curve analysis showed that at a depth of 3,000 and 500 sequence reads, the majority of the bacterial and fungal diversity was captured, respectively (Fig. S1).

### Composition of the healthy oral bacterial and fungal microbiota.

**FIG 2:**
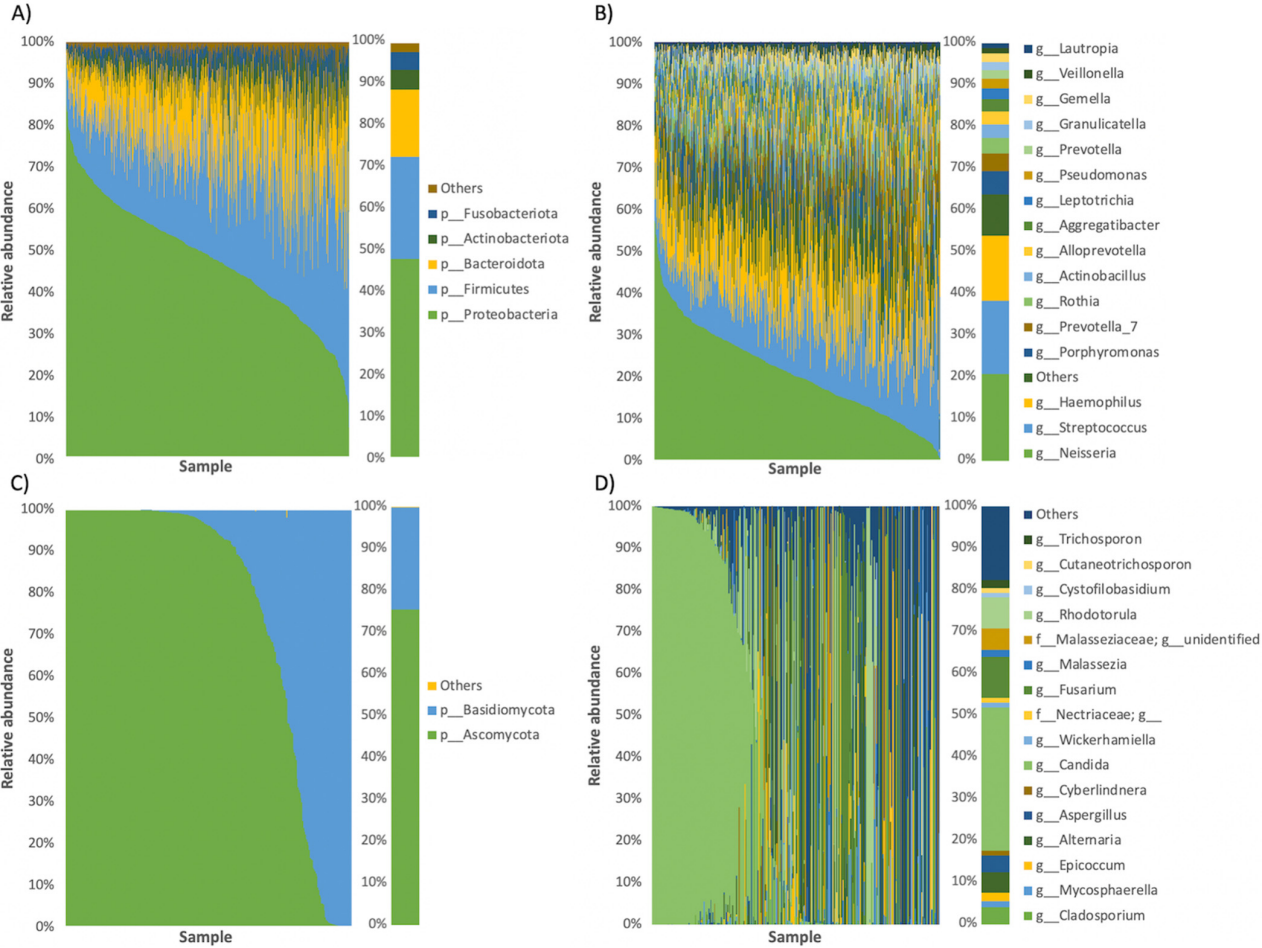
Taxonomic barplots of the bacterial (A-B) and fungal (C-D) oral microbiome at the phylum (A, C) and genus (B, D) levels. Taxa with a mean relative abundance < 1% were grouped into “Others”. The left panels show the results of each individual, whereas the right panels are the averaged values.

The oral bacterial microbiota in our healthy Chinese adult population was dominated by the phyla Proteobacteria (47.9%), Firmicutes (24.6%), Bacteroidota (16.2%), Actinobacteriota (4.8%), and Fusobacteriota (4.2%) (Fig. 2A). Top bacterial genera included *Neisseria* (20.8%), Streptococcus (17.4%), Haemophilus (15.6%), *Porphyromonas* (5.5%), and *Prevotella_7* (4.2%) (Fig. 2B). Using a prevalence threshold of 90% and 70%, 19 core genera and 11 core ASVs were identified, respectively (Data set S1 in the supplemental material). In particular, the genera *Neisseria*, Streptococcus, Haemophilus, *Leptotrichia*, and *Granulicatella* were detected in all 664 samples and made up an average of 58.4% of the total microbial community. Core ASVs included those identified as Haemophilus parainfluenzae T3T1, Rothia mucilaginosa, Rothia aeria, and Rothia dentocariosa ATCC 17931.

The oral fungal microbiota in our healthy population was composed of the phyla Ascomycota (75.5%) and Basidiomycota (24.5%) (Fig. 2C). It was most represented by the genus *Candida* (34.3%), followed by Fusarium (9.7%), *Rhodotorula* (7.5%), *Alternaria* (5.0%), and *Cladosporium* (4.1%) (Fig. 2D). Among all genera, *Candida* appeared in 65% of all 304 samples, representing a core genus of the healthy oral fungal microbiota.

### Determinants of alpha diversity of the healthy oral microbiota.

**FIG 3:**
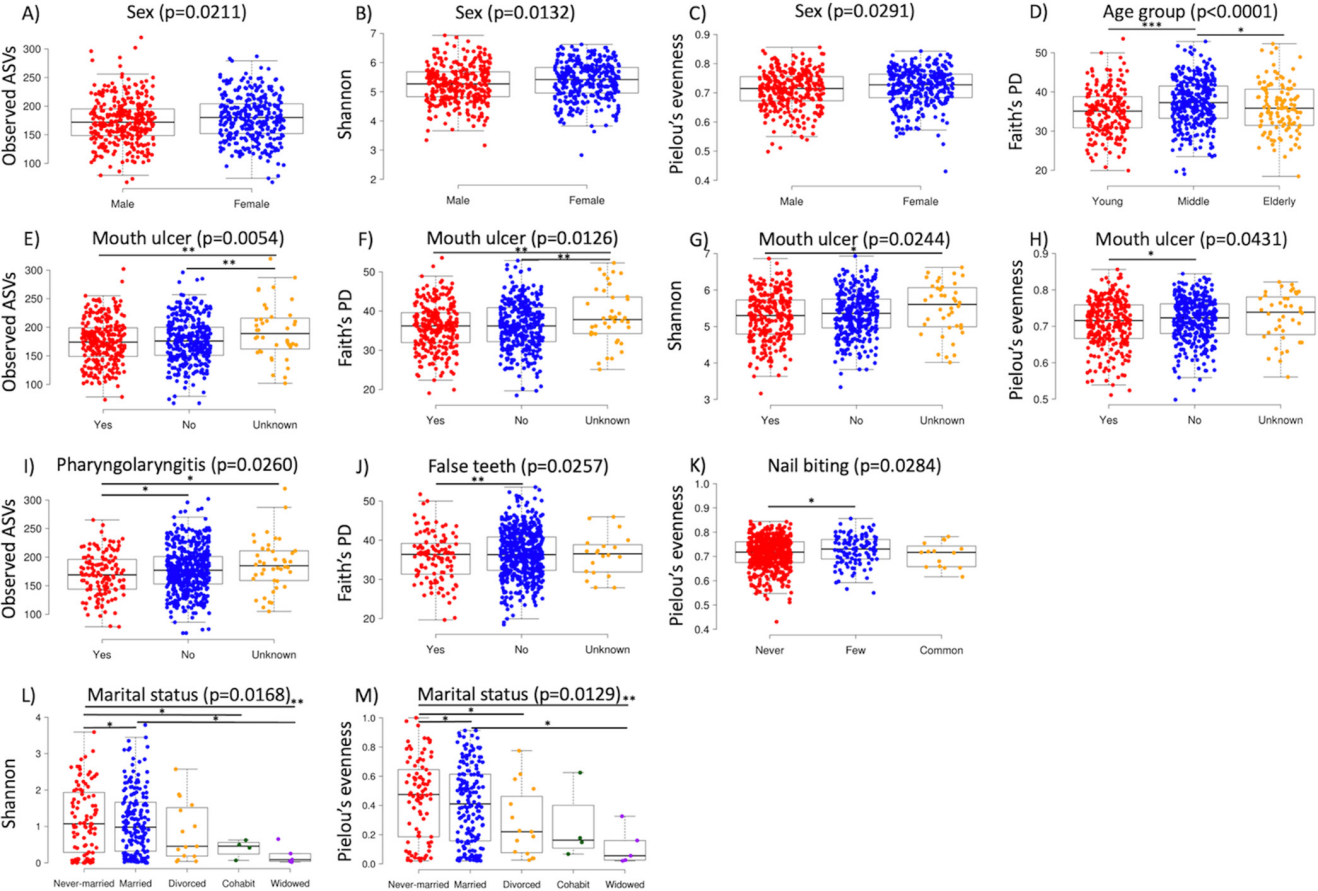
Boxplots showing the associations between metadata variables and alpha diversity in the bacterial (A–K) and fungal (L–M) oral microbiome. Apart from sex and age group, all other variables were controlled for sex and age. Only statistically significant variables are shown here. *, *P < *0.05; **, *P < *0.01; ***, *P < *0.001.

Among the 24 metadata variables examined, sex was significantly associated with the number of observed ASVs, Shannon diversity and Pielou’s evenness of the healthy oral bacterial microbiome (*P < *0.05), whereas age was significantly associated with Faith’s PD (*P < *0.001, *q *< 0.001) (Fig. 3; Data set S2 in the supplemental material). After controlling for sex and age, significant associations were observed between alpha diversity metrics and nail-biting and recent oral conditions, namely, false teeth, mouth ulcer, and pharyngolaryngitis within 3 months (*P < *0.05); however, none of them remained significant after controlling for multiple comparisons (*q *> 0.1) (Fig. 3; Data set S2).

Unlike in the case of bacterial microbiome, no significant associations were observed between sex or age and any of the alpha diversity metrics calculated for the oral mycobiome (*P > *0.05) (Data set S3). Among all 24 metadata variables examined, only marital status was observed to be associated with Shannon diversity and Pielou's evenness of the healthy oral mycobiome (*P < *0.05). The associations remained significant after controlling for sex and age (*P < *0.05) but not multiple comparisons (*q *> 0.1) (Fig. 3; Data set S3).

### Determinants of beta diversity of the healthy oral microbiota.

**FIG 4:**
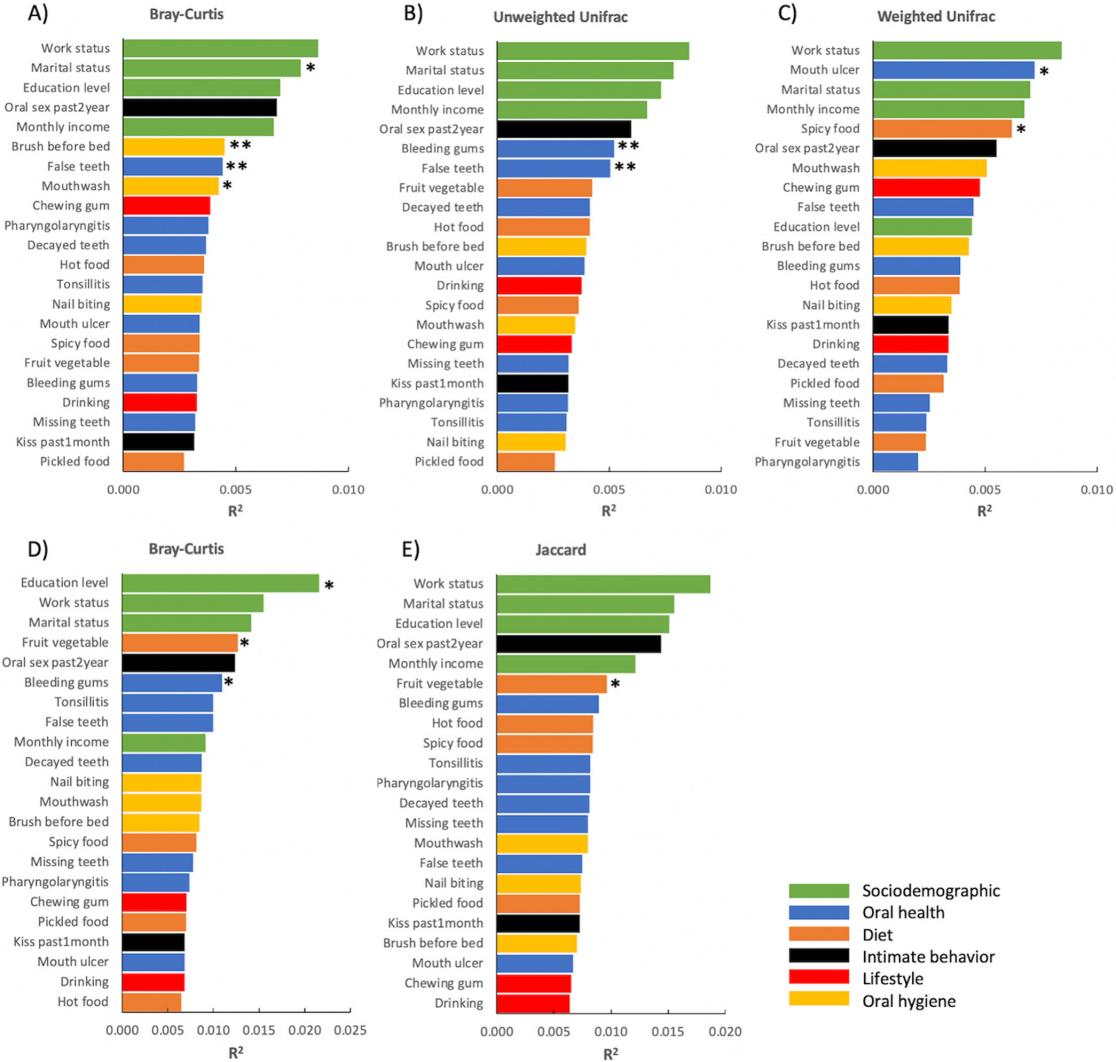
Effect size of metadata variables on the oral bacterial (A–C) and fungal (D–E) microbiota composition based on Bray–Curtis dissimilarity (A, D), unweighted Unifrac (B), weighted Unifrac (C), and Jaccard (E) distances after controlling for sex and age. Bars are colored based on the category of the variables. *, *P < *0.05; **, *P < *0.01.

Similar to alpha diversity, sex and age were significantly associated with all three beta diversity metrics calculated for the oral bacterial microbiome (*P < *0.01, *q *< 0.1) (Fig. S2, Data set S4 in the supplemental material). Age had a relatively large effect on the overall structure of the bacterial microbiome based on all three beta diversity metrics (Bray–Curtis, *R*^2^ = 0.0066; unweighted UniFrac, *R*^2^ = 0.0102; weighted UniFrac, *R*^2^ = 0.0096); however, a large effect of sex was only observed in the weighted UniFrac distance metric (Bray–Curtis, *R*^2^ = 0.0029; unweighted UniFrac, *R*^2^ = 0.0032; weighted UniFrac, *R*^2^ = 0.0077) (Fig. S3). After controlling for sex and age, largest impacts on the healthy oral bacterial microbiome were attributed to marital status (*R*^2^ = 0.0079, *P < *0.05), oral hygiene-related variables, namely, brush before bed (*R*^2^ = 0.0045, *P < *0.01) and mouthwash (*R*^2^ = 0.0042, *P < *0.05), and false teeth (*R*^2^ = 0.0044, *P < *0.01) based on Bray–Curtis dissimilarity; recent oral conditions, namely, bleeding gums (*R*^2^ = 0.0052, *P < *0.01) and false teeth (*R*^2^ = 0.0051, *P < *0.01) within 3 months based on unweighted UniFrac distance; and mouth ulcer (*R*^2^ = 0.0072, *P < *0.05) and spicy food (*R*^2^ = 0.0062, *P < *0.05) based on weighted UniFrac distance (Fig. 4A to C, Data set S4). Significant associations remained after controlling for multiple comparisons included those between Bray–Curtis dissimilarity and brush before bed and false teeth, and those between unweighted UniFrac distance and false teeth and bleeding gums (*q *< 0.1).

For fungi, beta diversity analysis revealed that both sex and age were significantly associated with Jaccard distance (*P < *0.05, *q *< 0.1); however, for Bray–Curtis dissimilarity, significant association was only observed in age without controlling for multiple comparisons (*P < *0.05, *q *> 0.1) (Fig. S4, Data set S5 in the supplemental material). Unlike in the case of bacterial microbiome, age only showed a relatively small effect (*R*^2^ < 0.0080) based on both beta diversity metrics (Fig. S5). After controlling for sex and age, largest impacts on the healthy oral mycobiome were attributed to education level (*R*^2^ = 0.0216, *P < *0.05), followed by fruits and vegetables (*R*^2^ = 0.0127, *P < *0.05) and bleeding gums (*R*^2^ = 0.0110, *P < *0.05) based on Bray–Curtis dissimilarity; and fruits and vegetables (*R*^2^ = 0.0096, *P < *0.05) based on Jaccard distance (Fig. 4D and E); however, none of them remained significant after controlling for multiple comparisons (*q *> 0.1) (Data set S5 in the supplemental material).

### Differentially abundant bacterial and fungal ASVs across metadata groups.

**FIG 5:**
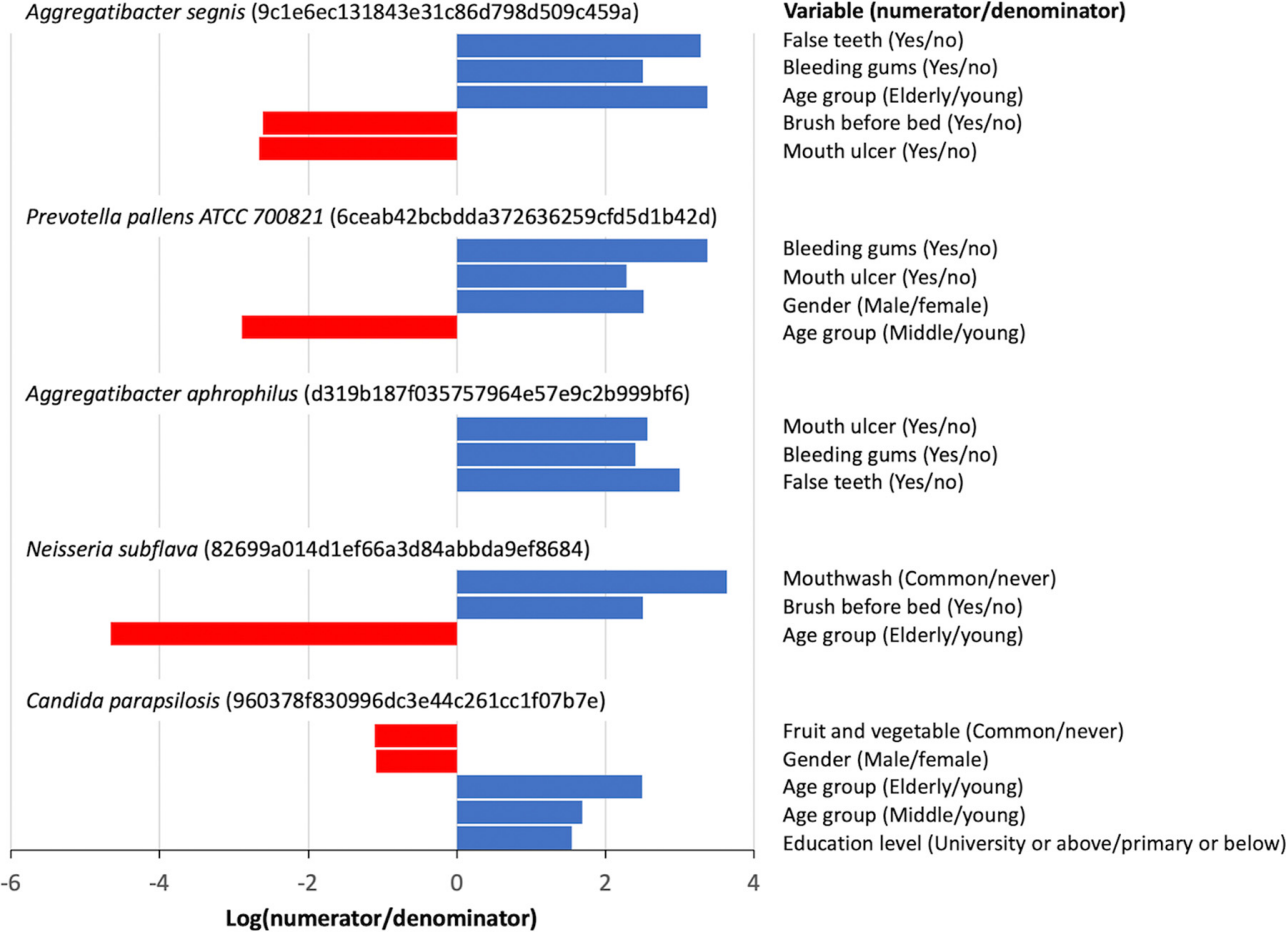
Examples of differentially abundant ASVs as detected by Songbird. Only ASVs that can be identified to the species level and were differentially abundant across multiple metadata variables are shown here. ASV IDs are provided after each species name. For each ASV, a positive log(numerator/denominator) ratio for a particular metadata variable (blue bar) indicates an enrichment of the ASV in the numerator group compared to the denominator group, whereas a negative ratio (red bar) indicates an enrichment in the denominator group.

Using the compositionality-aware tool Songbird, differentially abundant bacterial ASVs were identified between groups in nine metadata variables (Data set S6 in the supplemental material). Some ASVs were associated with multiple metadata variables. For instance, an ASV belonging to Aggregatibacter segnis was enriched in individuals with false teeth or bleeding gums or in the elderly and was depleted in those who brush before bed or with mouth ulcer (Fig. 5). Another ASV identified as Aggregatibacter aphrophilus was more abundant in those with mouth ulcer, bleeding gums or false teeth. Besides, an ASV belonging to Prevotella pallens was enriched in individuals with bleeding gums or mouth ulcer or in male and was depleted in those in middle age. Finally, an ASV identified as Neisseria subflava was more abundant in those who commonly use mouthwash or brush before bed and was less abundant in the elderly.

Similarly, differentially abundant fungal ASVs were identified between groups in four metadata variables (Data set S7 in the supplemental material). For bleeding gums, which also showed significant association in the beta diversity analysis, a Songbird Q^2^ value < 0 was obtained, indicating poor predictive accuracy and suggesting possible overfitting of model, and was thus removed from the analysis. Similar to the case of bacterial microbiome, some fungal ASVs were associated with multiple metadata variables. Example includes an ASV belonging to Candida parapsilosis, which was depleted in individuals who commonly consume fruits and vegetables or in male and enriched in the elderly or middle age or with a high education level (Fig. 5).

### Bacterial–fungal interactions in the healthy oral microbiota.

High-quality 16S and ITS sequence data were available from 292 healthy individuals (Fig. 1). These data sets were used to study associations between the oral bacterial and fungal microbiota in healthy individuals. Bacterial–fungal associations were first examined at the community level based on alpha and beta diversity. No association was observed between the alpha diversity of the bacterial and fungal microbiomes (number of observed ASVs, Spearman’s ρ = -0.01, *P = *0.91; Shannon diversity, Spearman’s ρ = -0.01, *P = *0.89; Pielou’s evenness, Spearman’s ρ = -0.02, *P = *0.70) (Fig. S6 in the supplemental material). Similarly, no association was observed between the beta diversity of the bacterial and fungal microbiomes (Bray–Curtis, Spearman’s ρ = 0.04, *P = *0.10; Jaccard, Spearman’s ρ = 0.04, *P = *0.04) (Fig. S7).

**FIG 6:**
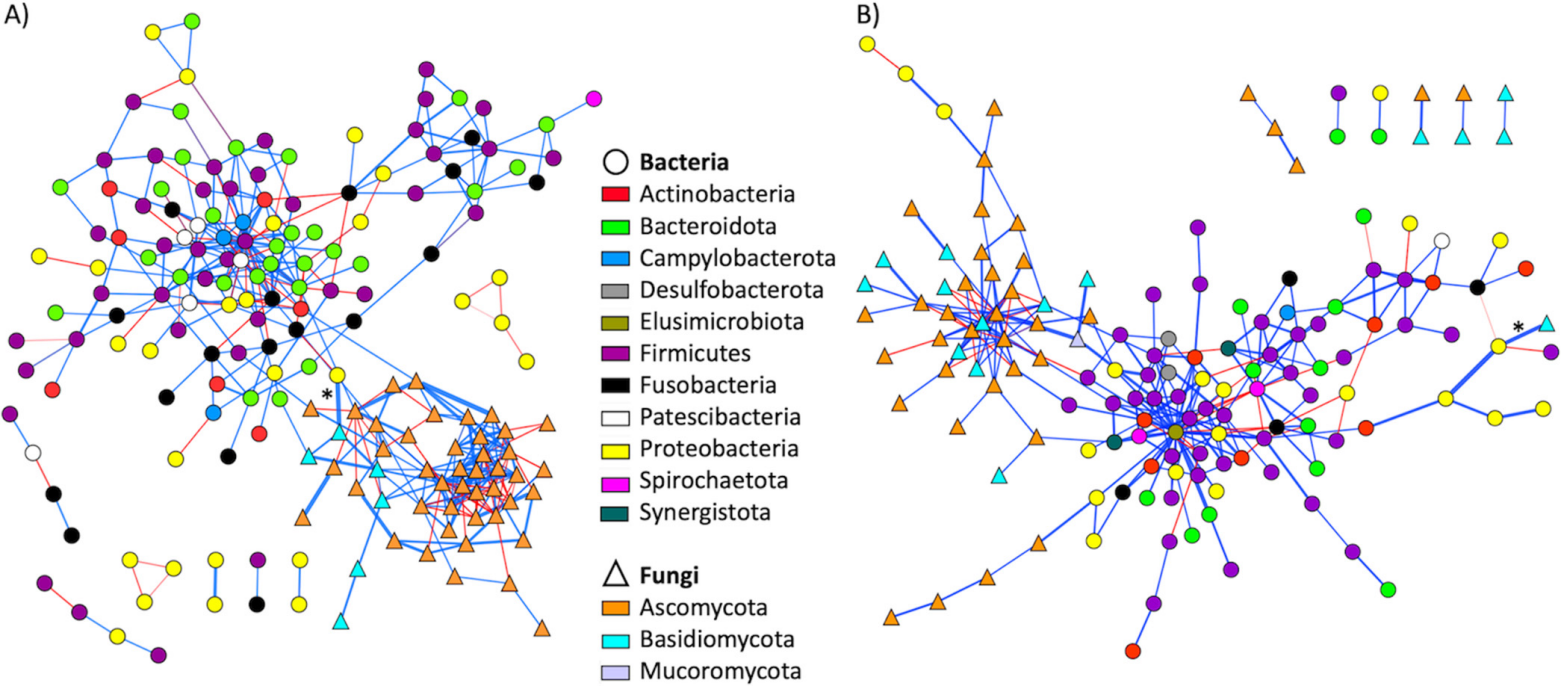
Cross-kingdom association networks at the amplicon sequence variant (ASV) (A) and genus (B) levels constructed using SPIEC-EASI. Taxa, as nodes, from different kingdoms are in different shapes and those from different phyla are in different colors. Edges in blue represent positive interactions, whereas those in red are negative ones. The thickness of edges are proportional to the interaction strength. The strongest bacterial–fungal interaction in each network is marked with an asterisk. Only connected nodes are shown here.

Bacterial–fungal interactions were then studied at the taxon level by constructing cross-kingdom association networks. The ASV-based network constructed was composed of 177 connected nodes, including 128 bacterial ASVs and 49 fungal ASVs (Fig. 6A). Forty-seven ASVs, 45 from bacteria and two from fungi, were unconnected and not shown in the final network. There were 383 interactions (as edges) in the network, 286 (74.7%) of which were positive and 97 (25.3%) of which were negative. The strongest positive interactions were mainly between fungal ASVs, whereas the strongest negative interactions were mainly between bacterial ASVs (Data set S8 in the supplemental material). Six interactions (1.57%) between bacterial and fungal ASVs were determined. All of them were positive but the strength of five of them were weak (edge weight < 0.05) (Data set S8). The strongest bacterial–fungal interaction (edge weight: 0.59) was observed between a bacterial ASV belonging to the genus Pseudomonas from the phylum Proteobacteria (prevalence: 16.72%, relative abundance: 2.21%) and a fungal ASV belonging to *Rhodotorula dairenensis* from the phylum Basidiomycota (prevalence: 16.61%, relative abundance: 6.90%) (Data set S8).

The association network constructed at the genus level comprised of 148 connected nodes, including 96 bacterial genera and 52 fungal genera (Fig. 6B). There were 263 interactions in the network, majority (83.7%) of which were positive. Ten interactions (3.80%) between bacterial and fungal genera were predicted, nine of which were positive (Data set S8 in the supplemental material). Strongest bacterial–fungal interactions were observed between Pseudomonas and *Rhodotorula* (edge weight = 0.48), *Roseburia* and *Rhizomucor* (edge weight = 0.29), *Citrobacter* and *Lasiodiplodia* (edge weight = 0.20), and Enterobacter and *Botrytis* (edge weight = 0.16).

### Potential keystone ASVs in the healthy oral microbiota.

**FIG 7:**
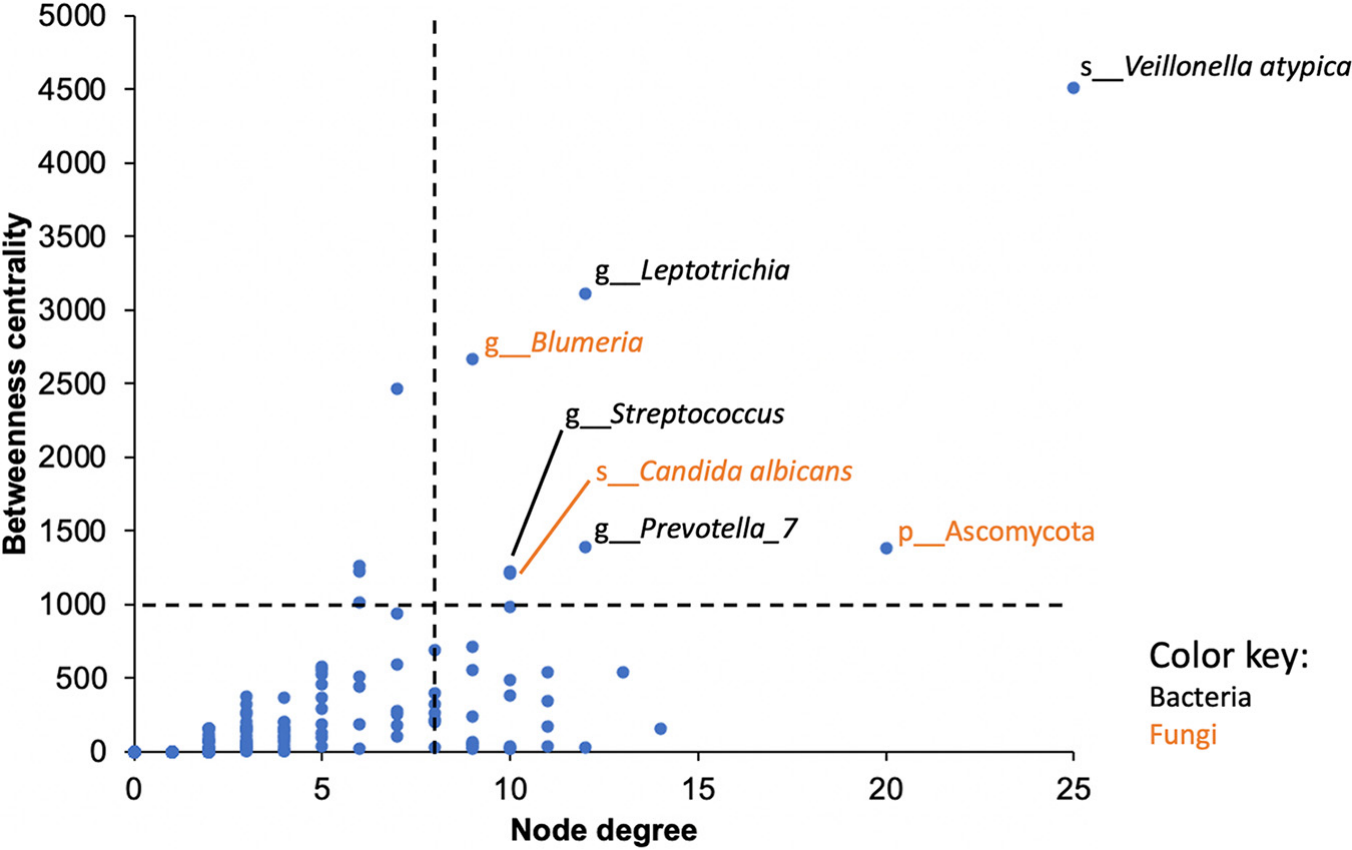
Keystone species analysis. Potential keystone ASVs were identified as those with a high node degree (>8) and betweenness centrality (>1,000) in the association network. Four potential bacterial keystone ASVs and three potential fungal keystone ASVs were identified and labeled here. The dashed lines correspond to the cutoff values used here.

Potential keystone ASVs were identified based on their degree and betweenness centrality in the network. Seven ASVs had a degree > 8 and betweenness centrality > 1,000 and were regarded as potential keystone ASVs here (Fig. 7, Table S2 in the supplemental material). These included four bacterial ASVs, belonging to Veillonella atypica and the genera *Leptotrichia*, *Prevotella_7* and Streptococcus, and three fungal ASVs, belonging to Candida albicans, genus *Blumeria*, and phylum Ascomycota.

## DISCUSSION

Although there are numerous studies on the healthy oral bacterial microbiome, our current understanding of the determinants of the healthy oral mycobiome remains limited, let alone knowledge on the bacterial–fungal interactions. In this study, we characterized the variations of the oral bacterial microbiome in 664 individuals and the oral mycobiome in 304 individuals of a systemically healthy Chinese population, examined their associations with a range of, mostly oral-related, metadata variables, and explored potential bacterial–fungal interactions and identified potential keystone species in the oral microbiome. To the best of our knowledge, this study represents one of the first population-based studies that characterized simultaneously the healthy oral bacterial and fungal microbiomes in the same cohort. It is also one of the largest studies on a healthy Chinese population (28, 29).

The oral bacterial microbiome in our healthy Chinese population was dominated by the genus *Neisseria*. This differs from findings from other populations. For instance, *Veillonella* dominates the salivary bacterial microbiome of a Canadian population (7), whereas *Prevotella* dominates that of a Qatari population (6). This points to a population-specific healthy oral bacterial microbiome, which may be attributed to factors such as host genetics and diet. However, genera such as Neisseria, Streptococcus, and Haemophilus were detected as core genera in all these studies as well as in ours, suggesting the presence of a global core bacterial microbiome in heathy adults. Additional studies on other underexplored populations are needed to verify this hypothesis. Compared with the bacterial counterpart, studies of the healthy oral mycobiome at the population level are scarce. Zakaria et al. (30) studied the salivary mycobiome in a healthy Japanese population of community-dwelling elderly and revealed the dominance of Candida albicans in most samples. *Candida* is also reported to be the predominant genus in the oral mycobiome of an American population comprising HIV-positive and HIV-negative individuals (31). The dominance of *Candida* in the oral mycobiome was also observed in our population, indicating its importance as a major member of the healthy oral mycobiome. However, as this most prevalent fungal genus revealed in our study was only detected in less than 70% of all individuals examined, it is unlikely for it, and other fungi alike, to represent a global core genus in the healthy oral mycobiome.

We examined the associations between 24 metadata variables related to sociodemographic characteristics, lifestyle, diet, oral hygiene, oral health, and oral intimate behavior and the oral bacterial and fungal microbiomes. We showed that sex and age were significantly associated with the structure of the oral bacterial microbiome, in agreement with findings from a healthy Canadian population (7). After controlling for sex and age, significant variables affecting the oral bacterial microbiome included marital status, oral hygiene-related variables, namely, brush before bed and mouthwash, recent oral conditions, namely, bleeding gums, false teeth, and mouth ulcer within 3 months, as well as spicy food. Marital status has been shown to associate with the taxonomic abundance of the oral bacterial microbiota in adult residents of New York City (32). Tooth brushing does not affect the bacterial community structure of subgingival plaque (33); however, tooth brushing frequency affects the relative abundance of periodontal bacteria in patients with intracranial aneurysms (34). Use of chlorhexidine mouthwash is associated with major shifts in the salivary bacterial microbiome (35). Bleeding gums, mouth ulceration and denture use have been reported to affect the salivary bacterial microbiome of a Qatari population (6). Although there is no specific report on the association of a spicy food diet on the oral microbiome, multiple studies have revealed a role of diet on the oral bacterial microbiome (36, 37). Overall, results from our current study are in concordance with previous reports, showing that the healthy oral bacterial microbiome is associated with marital status, oral hygiene, oral health, and diet.

Sex and age also significantly affected the structure of the oral mycobiome in our study population. However, unlike in the case of bacterial microbiome, age only showed a small effect compared to the other variables, indicating a smaller influence of it on the oral mycobiome than on the bacterial microbiome. After controlling for sex and age, significant variables affecting the oral mycobiome included education level, fruits and vegetables, and bleeding gums, in decreasing order of importance. Although information on the relationship between diet and oral mycobiome is lacking, several studies have revealed a role of diet on the gut mycobiome (reviewed in Ref. 38). Besides, research has shown that postpartum females with gingivitis, a symptom of which is bleeding gums, have altered abundance of numerous fungal genera compared to those with good oral health (39). The association between one's education level and their oral mycobiome is less clear; it is likely that a university degree may represent a composite variable of multiple factors that affect the oral mycobiome, including the choice of diet (40). While bleeding gums was also an explanatory variable for the oral bacterial microbiome, the other two were not. Besides, unlike in the case of bacterial microbiome, none of the oral hygiene-related variables were associated with the oral mycobiome. The presence of a different set of explanatory variables for the oral bacterial and fungal microbiomes suggests that the two microbial communities are shaped by different factors. However, it is noteworthy that while we focused mainly on oral-related variables in this study, the list of included variables is not exhaustive and has likely missed variables that might explain the variation of the microbiomes, such as fat-free mass (7) and flossing habit (5). In agreement with our findings, a study on the oral mycobiome of a population comprising HIV-positive and HIV-negative individuals based on ITS2 sequencing has identified sex as a significant variable affecting the oral mycobiome, with brushing frequency, mouthwash usage, frequency of alcoholic beverage intake, and frequency of oral sex being some of the insignificant variables (31). However, in contrast to our findings, the oral mycobiome in that study was also affected by caries status and missing teeth, and not by gingivitis and diet. Discrepancies in these findings could be due to differences in the study populations, sampling methods, design of questionnaire, choice of ITS regions, and/or bioinformatics approaches used.

We identified several ASVs that were differentially abundant in the oral microbiome across multiple metadata variables. A bacterial ASV belonging to Aggregatibacter segnis was found enriched in individuals with bleeding gums or false teeth, in the elderly, or in those who do not brush before bed. This is in concordance with previous suggestion of a potential etiological role of *A. segnis*, formerly Haemophilus segnis, in adult periodontitis (41). Another ASV belonging to *A. aphrophilus* was found more abundant in individuals with mouth ulcer, bleeding gums or false teeth. This supports previous suggestion that *A. aphrophilus* is a causative agent of periodontal disease (42). An ASV belonging to Prevotella pallens was enriched in individuals with bleeding gums or mouth ulcer, in male, or in young individuals. Although there are no previous reports on the association between *P. pallens* and bleeding gums or mouth ulcer, this bacterium together with Streptococcus mutans form a saliva-based diagnosis model for childhood caries (43). In contrast to the aforementioned potentially pathogenic oral bacteria, an ASV identified as generally nonpathogenic Neisseria subflava was found more abundant in those who commonly use mouthwash or brush before bed, or in young individuals (44). Regarding the fungal members, an ASV belonging to Candida parapsilosis was found enriched in the elderly and depleted in individuals who commonly consume fruits and vegetables. This agrees with previous reports on the increased *Candida* colonization in the oral cavity of the elderly (45). Our finding further suggests that regular consumption of fruits and vegetables may reduce C. parapsilosis colonization.

Bacterial–fungal interactions in the oral cavity of healthy individuals have been examined previously (46). However, the majority, if not all, of these studies predict potential interactions based on traditional statistical metrics such as Spearman's correlation. Since microbiome data are compositional in nature, these approaches can yield spurious results (26). Here, we studied the associations between the oral bacterial and fungal microbiota in healthy Chinese adults using SPIEC-EASI, a compositionally robust inference approach of cross-domain associations (11), based on 16S and ITS sequence data from 292 individuals. We showed no significant associations between the bacterial and fungal microbiota at the broad scale in terms of alpha and beta diversity. We then built cross-kingdom association networks to study bacterial–fungal interactions at the ASV and genus levels. Results showed that the majority (96%) of the fungal ASVs were connected to at least one bacterial/fungal member and that strongest positive interactions were mainly observed between fungal ASVs. These findings support previous postulations that fungi play a stabilizing role in the whole microbial community organization, as revealed by increased overall connectivity and stability in networks inferred for the human lung and skin microbiomes based on bacteria and fungi combined compared to those based on either domain (11). Cross-kingdom interactions made up only 1.6% and 3.8% of the total interactions at the ASV and genus level, respectively, in the healthy oral microbiome. These values are comparable to that (4.5%) obtained for the human lung microbiome (11) and indicate limited bacterial–fungal interactions in the oral microbiome of healthy adults.

Among the cross-kingdom interactions predicted, the strongest association was observed between a bacterial ASV belonging to the genus Pseudomonas and a fungal ASV belonging to Rhodotorula dairenensis. A strong association between Pseudomonas and Rhodotorula was also detected in the genus-level association network. Pseudomonas is a diverse and metabolically versatile genus of bacteria. While Pseudomonas members are not normally considered inhabitants of the healthy oral cavity, they can be enriched in individuals with oral conditions such as chronic periodontitis (47) or receiving orthodontic treatment (48). Rhodotorula is a dominant fungal genus in the oral cavity of healthy individuals. Although R. dairenensis has been detected in clinical blood culture samples (49), there is no report of its presence in the oral cavity, in particular of healthy individuals, in the literature. The high edge weight and relative abundance of both ASVs, and the low prevalence of both ASVs indicate that the Pseudomonas–R. dairenensis interaction is a strong cross-kingdom interaction between dominant ASVs of the oral microbiome that appears in a small subset of healthy individuals, possibly with undesirable oral health conditions. However, the inability to identify the Pseudomonas ASV down to the species level has hindered interpretation of the importance of the interaction from a biological perspective. Nonetheless, coculture experiments should be performed in the future to validate the predicted interactions (11).

Keystone species are species which have a disproportionately large effect on an ecosystem relative to their abundance (50). Here, four bacterial ASVs and three fungal ASVs were identified as potential keystone taxa of the healthy oral microbiota based on network analysis. An ASV belonging to Veillonella atypica had the highest degree and betweenness centrality, a low relative abundance and high prevalence, representing a strong candidate of keystone taxa. V. atypica and other Veillonella species are early colonisers in oral biofilm formation (51). They are suggested to play a key role in maintaining oral health due to their ability to produce nitrite from nitrate and convert lactic acid to weaker acids and thereby preventing oral diseases (52). Another potential keystone taxon detected here is an ASV belonging to the genus Streptococcus. Commensal Streptococcus species are initial colonisers of the oral cavity. They are considered key players in oral homeostasis due to their metabolic plasticity and abilities to colonize multiple oral surfaces, moderate biofilm acidification, generate hydrogen peroxide, and secrete antimicrobial compounds (53). An ASV belonging to the genus Prevotella_7 represents another potential keystone taxon. Prevotella_7 is a genus-level subdivision of the diverse genus Prevotella designated based on the degree of sequence divergence and includes species such as P. multiformis, P. albensis, and P. dentalis (54). Previous physical proximity analysis has revealed that members of the genus Prevotella, together with those of Actinomyces, display extensive inter-generic associations, suggesting a central role of them in establishing and maintaining oral biofilm complexity (55). However, despite their abundance and role in oral biofilm formation, Prevotella species in the oral cavity are understudied compared with other oral bacteria (56). The last potential bacterial keystone taxon detected here is an ASV belonging to the genus Leptotrichia. Leptotrichia species are primarily oral commensals but are also opportunistic pathogens (57). It has been reported that Leptotrichia species can trigger the transcription levels of both pro- and anti-inflammatory interleukins in oral epithelial cells, suggesting their crucial role in the “fine-tune” regulation of epithelial immune response (58).

Candida albicans is among one of the fungal keystone taxa identified in the healthy oral microbiota here. Based on reported physical, metabolic, and chemical interactions between C. albicans and a range of bacteria in the oral cavity, it has been argued that C. albicans should be regarded as a keystone commensal in the oral cavity (59). Young et al. (60) tested this hypothesis using *in vitro* polymicrobial oral biofilm models, showing that the presence of C. albicans increased the level of metabolic activity and biomass of bacteria in the biofilms. An ASV belonging to the genus Blumeria represents another potential fungal keystone taxon in the oral microbiome of healthy adults. An unidentified species of Blumeria has also been detected in high abundance in dental plaque of caries-free children (61). However, additional information of Blumeria species in the oral cavity are scarce to date. Overall, results of our current network analysis are in concordance with previous reports on the importance of well-known oral microbes, such as Streptococcus species and C. albicans, providing further support to their potential pivotal roles in the oral microbiome of healthy adults. Our results have also unveiled potential important roles of understudied taxa, such as Prevotella_7 and Blumeria, in the healthy oral microbiome, providing clues for future research directions on this subject.

There are several limitations to our study. First, data on sampling time of day (62), medication use (63), and anthropometric measurements such as fat-free mass and waist-hip ratio (7), all of which have been reported to affect the oral microbiome, are not available in our study. Second, most metadata collected in this study were self-reported. Lastly, the use of the 16S rRNA gene V1-V2 region, just like other regions, hinders accurate taxonomic identification down to the species or strain level (64).

## CONCLUSIONS

To conclude, in this study, we characterized simultaneously for the first time the oral bacterial and fungal microbiomes in a large cohort of healthy Chinese adults. We examined the associations between 24 metadata variables and the oral microbiomes and showed that the oral bacterial and fungal microbiomes in healthy Chinese adults are shaped by a different set of factors. By performing network analysis, we suggest that bacterial–fungal interactions are limited in the healthy oral microbiome. Besides, we have identified Veillonella atypica and Candida albicans, among a few other taxa, as potential keystone taxa of the healthy oral microbiome. Overall, our study has facilitated understanding of the determining factors and cross-kingdom interactions of the healthy human oral microbiome. Future studies should validate interactions predicted here using techniques such as coculture.

## MATERIALS AND METHODS

### Study population.

The current study includes the analysis of a subset of oral rinse samples collected from our previous population-wide oral human papillomavirus (HPV) screening study in Hong Kong (12). In the original study, a cohort of 1,469 local residents were recruited through media advertisements and health talks in the community from 2015 to 2016. Each participant completed a questionnaire including information on sociodemographic characteristics, lifestyle, diet, oral hygiene, oral health, and oral intimate behavior (Table S1 in the supplemental material). The inclusion criteria for the current study were ethnically Chinese aged  ≥ 18 years at the time of recruitment. Current smokers and subjects with diagnosis of cancer, diabetes, hypertension or sexually transmitted diseases, drug eruption, tonsillectomy, lichen planus, pemphigus, oral leukoplakia, Behcet's disease or erythema multiforme in the last 3 months, long-term use of steroid, and infection with high-risk HPV were excluded to compose a cohort of healthy subjects.

### Sample collection, DNA extraction, and amplicon sequencing.

Oral rinse samples were collected in 20 ml 0.9% normal saline gargled twice, for 20 and 10 s. Approximately 1 ml of the oral rinse solution was centrifuged at 5,000 g for 5 min and DNA was extracted from the pellet using the QIAamp DNA Mini-Kit (Qiagen, Germany) following the manufacturer’s instructions. The bacterial 16S rRNA gene V1-V2 region was amplified with PCR using universal primers 27F-YM (5′-AGA GTT TGA TYM TGG CTC AG-3′) and 338R (5′-TGC TGC CTC CCG TAG GAG T-3′), whereas the fungal internal transcribed spacer 1 (ITS1) region was amplified with ITS1F (5′-CTT GGT CAT TTA GAG GAA GTA A-3′) and ITS2 (5′-GCT GCG TTC TTC ATC GAT GC-3′). A pair of dual 12 bp barcodes was indexed to each amplicon set through the forward and reverse primers modified from the Earth Microbiome Project protocol (13). PCR products were pooled and sequenced on an Illumina MiSeq instrument (Illumina, San Diego, CA) at The Genomics Core Facility of the Weill Cornell Medicine Core Laboratories Centre following the 2 × 300 bp paired-end sequencing protocol. Negative controls, positive controls (mock communities), and technical replicates were also sequenced for quality control.

### Microbiome analysis.

Microbiome analysis was performed with QIIME2 2020.11 (14), unless specified otherwise. Primers were first trimmed from demultiplexed raw sequence data using the q2-cutadapt plugin (15). No insertions or deletions of bases were allowed when matching primers, and reads with no primer found were discarded. Paired-end reads were then joined using q2-vsearch (16), quality-filtered using q2-quality-filter, and denoised using q2-deblur (17). Taxonomy was assigned to amplicon sequence variants (ASVs) using Naïve Bayes classifiers trained on the V1-V2 region of the SILVA 138 SSU Ref NR 99 data set for 16S (18) and full-length UNITE ver. 8.3 dynamic data set for ITS (19) with q2-feature-classifier (20). ASVs with a total read count <10 or present in only one sample were removed. Archaeal, mitochondrial, chloroplast, and phylum-unclassified reads were also discarded. Samples with <3,000 or <500 quality-filtered sequence reads were removed from the 16S and ITS data sets, respectively. Feature tables were collapsed at different taxonomic levels using q2-taxa. Core bacterial ASVs and genera, defined here at a prevalence >70% and >90%, respectively, were identified using the core-features function of q2-feature-table.

Representative sequences of bacterial ASVs were inserted into the SILVA 128 SEPP reference phylogenetic tree with SATé-enabled phylogenetic placement (SEPP) using q2-fragment-insertion (21). After rarefying the samples to the smallest number of sequences, alpha and beta diversity metrics as well as principal coordinate analysis (PCoA) plots were generated using the q2-diversity plugin with the core-metrics-phylogenetic pipeline and core-metrics pipeline for 16S and ITS, respectively. Alpha diversity metrics computed for both 16S and ITS included the number of observed ASVs, Shannon diversity, and Pielou's evenness, whereas Faith’s phylogenetic diversity (PD) was also computed for 16S. Beta diversity metrics computed for 16S included Bray–Curtis dissimilarity and weighted and unweighted UniFrac distances, whereas Bray–Curtis dissimilarity and Jaccard distance were computed for ITS. Jaccard distance of 16S was additionally computed in the analysis of bacterial–fungal interactions. Alpha rarefaction curves were generated using the alpha-rarefaction function of q2-diversity. Associations between categorical metadata variables and alpha diversity metrics were tested using q2-diversity with the alpha-group-significance function. Individuals were binned into three age groups, namely, young (18–39 yr), middle (40–59 yr) and elderly (60 yr or above) (22). Samples with missing data in any metadata variable were removed and the effect size of each metadata variable was calculated using the adonis function of q2-diversity with 9,999 permutations. Sex- and age-controlled associations between metadata variables and sample composition were tested using the same function by adding sex and age into the model formula. Similarly, sex- and age-controlled associations between metadata variables and alpha diversity metrics were tested using the anova function of q2-longitudinal (23). For metadata variables showing significant difference in beta diversity, differentially abundant ASVs were identified using the compositionality-aware q2-Songbird plugin (24) in QIIME2 2019.7 while controlling for sex and age. Feature rankings and log-ratios produced were then visualized using q2-qurro (25).

### Network analysis.

Cross-kingdom association networks were built from samples with both high-quality 16S and ITS sequence data. Feature tables generated from QIIME2 were imported into R as phyloseq objects using the R package *qiime2R* ver. 0.99.6 (https://github.com/jbisanz/qiime2R). Rare bacterial ASVs appearing in less than 20% of samples and fungal ASVs appearing in less than 5% of samples were filtered. Cross-kingdom networks were then constructed using the R package *SpiecEasi* ver. 1.1.1 with the Meinshausen–Bühlmann neighborhood selection method (11, 26). The strength of associations between members in the network was estimated using edge weights, which are the average covariations from the estimated covariance matrix between all members in concern; a higher edge weight indicates a stronger association (11). Degree (the number of edges a node has) and betweenness centrality (proportion of the shortest paths in the network that pass through a node) of nodes in the networks were computed using the R package *igraph* ver. 1.2.6 (27). Potential keystone ASVs were identified as nodes with a high degree and betweenness centrality (11). Cross-kingdom association networks were also built using feature tables collapsed at the genus level.

### Statistical analysis.

Differences in alpha diversity among groups were tested using Kruskal–Wallis test, whereas differences in beta diversity were tested using permutational multivariate analysis of variance with 999 permutations. *P values* were adjusted using the Benjamini–Hochberg procedure to control for multiple comparisons. Beta diversity of the 16S and ITS data sets were compared using Mantel test in q2-diversity with 9,999 permutations. Differences were considered statistically significant when *P < *0.05 or *q *< 0.1.

### Data availability.

All sequence data generated from this study were deposited in the NCBI Sequence Read Archive under BioProject accession PRJNA778006.
