# Supplementary material for: Determinants and Interactions of Oral Bacterial and Fungal Microbiota in Healthy Chinese Adults
Source: Microbiol Spectr. 2022 Feb 2;10(1):e02410-21. doi: 10.1128/spectrum.02410-21 (PMC8809354; doi:10.1128/spectrum.02410-21)
Supplement: SUPPLEMENTAL FILE 1 — Supplemental material. Download SPECTRUM02410-21_Supp_1_seq8.pdf, PDF file, 4.2 MB [file spectrum02410-21_supp_1_seq8.pdf]

Supplementary figures

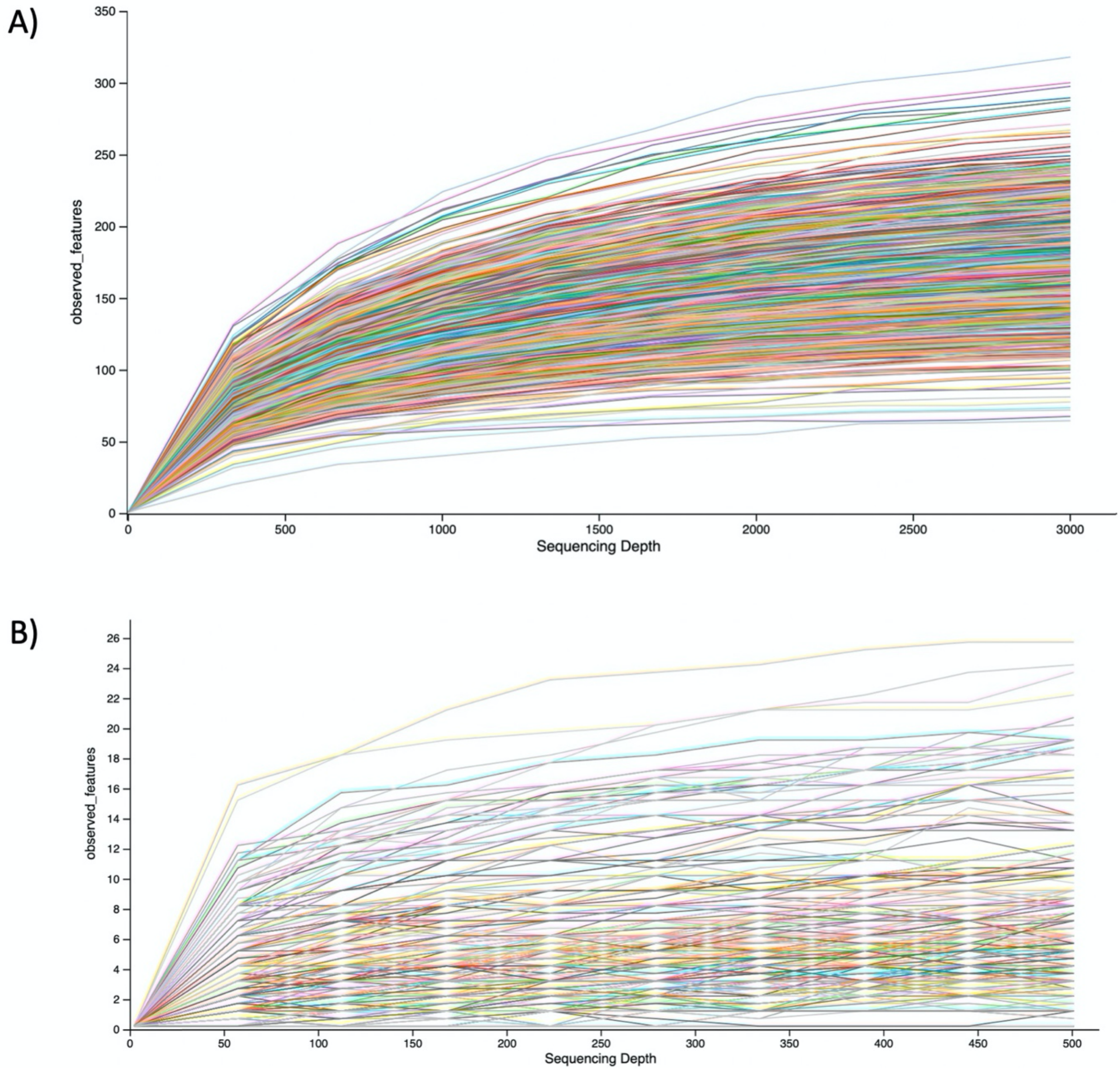

Figure S1. Rarefaction curves of the 16S rRNA (A) and ITS (B) sequence datasets.

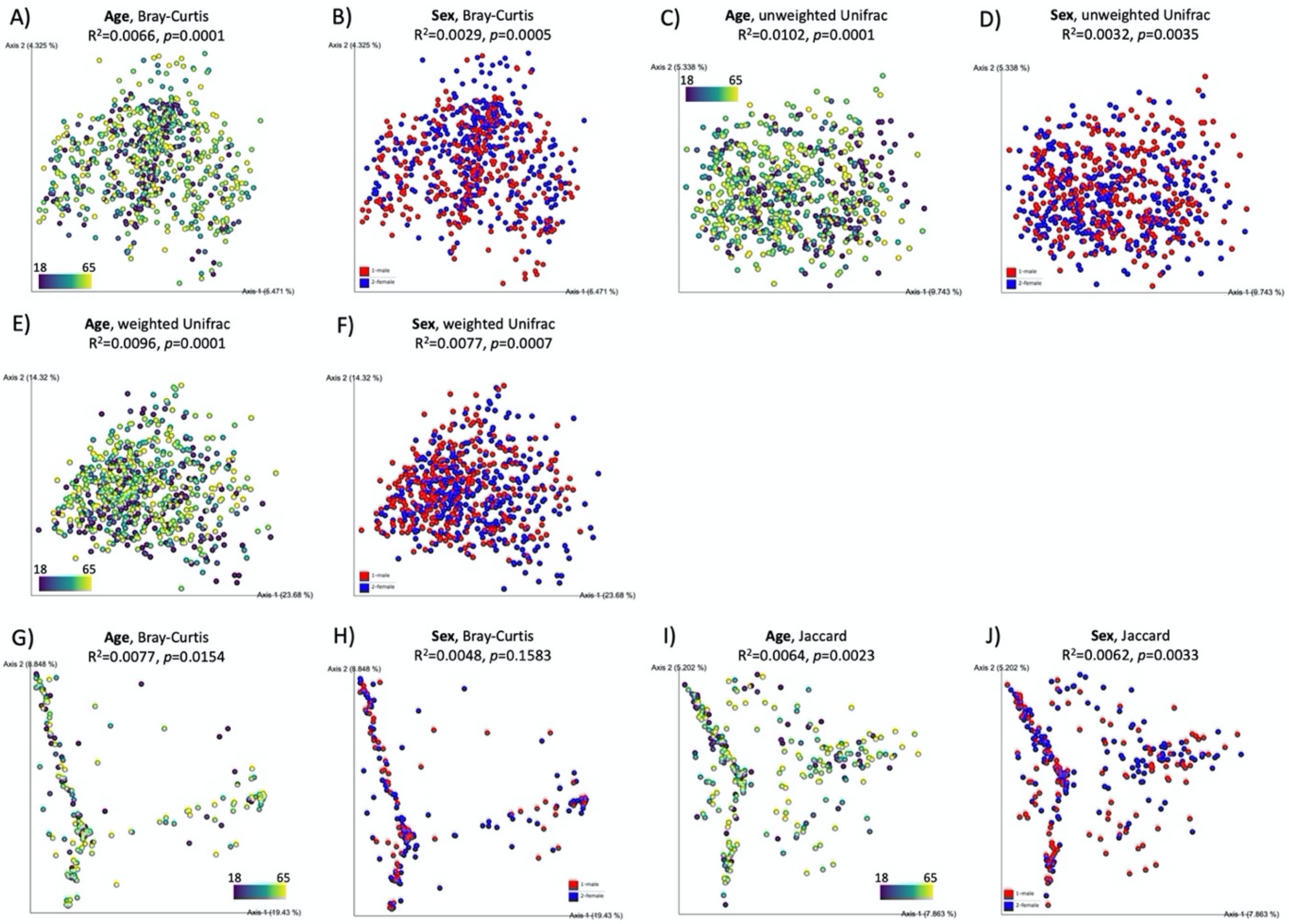

Figure S2. PCoA plots based on different distance metrics of the oral bacterial (A-F) and fungal (G-J) microbiome stratified by age and sex.

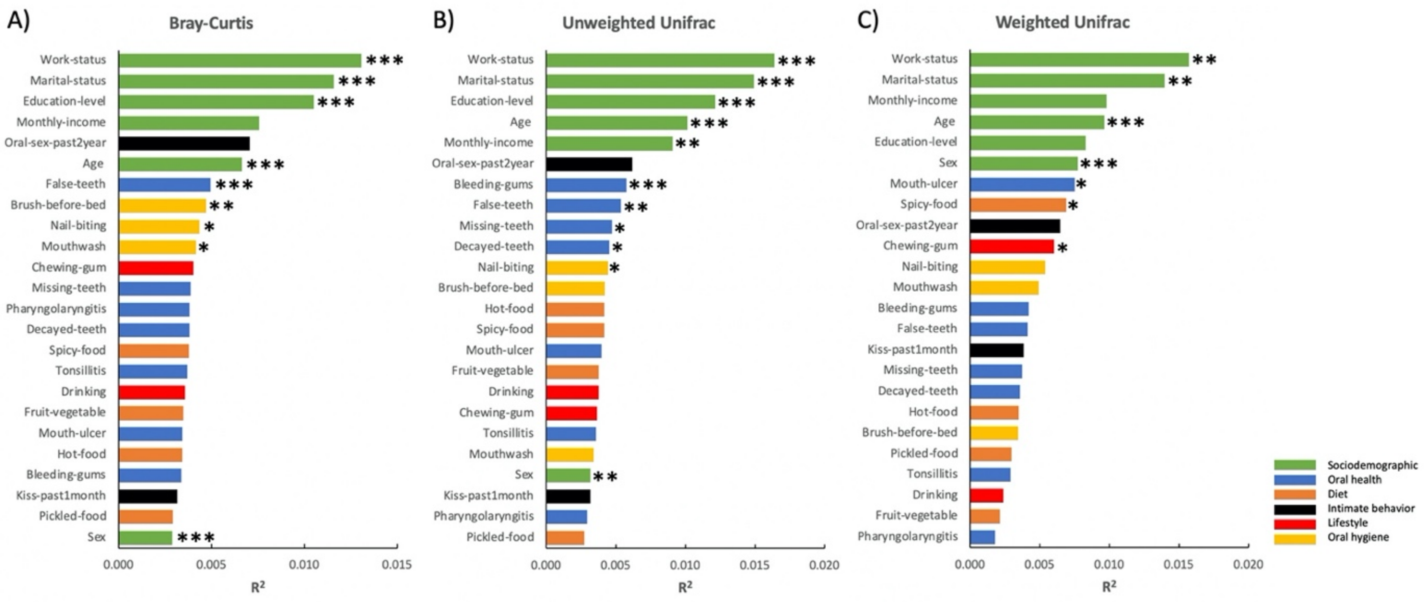

Figure S3. Effect size of metadata variables on the oral bacterial microbiota composition based on Bray–Curtis dissimilarity (A), unweighted Unifrac (B) and weighted Unifrac (C) distances. Bars are coloured based on the category of the variables. *$p < 0.05$, **$p < 0.01$, ***$p < 0.001$.

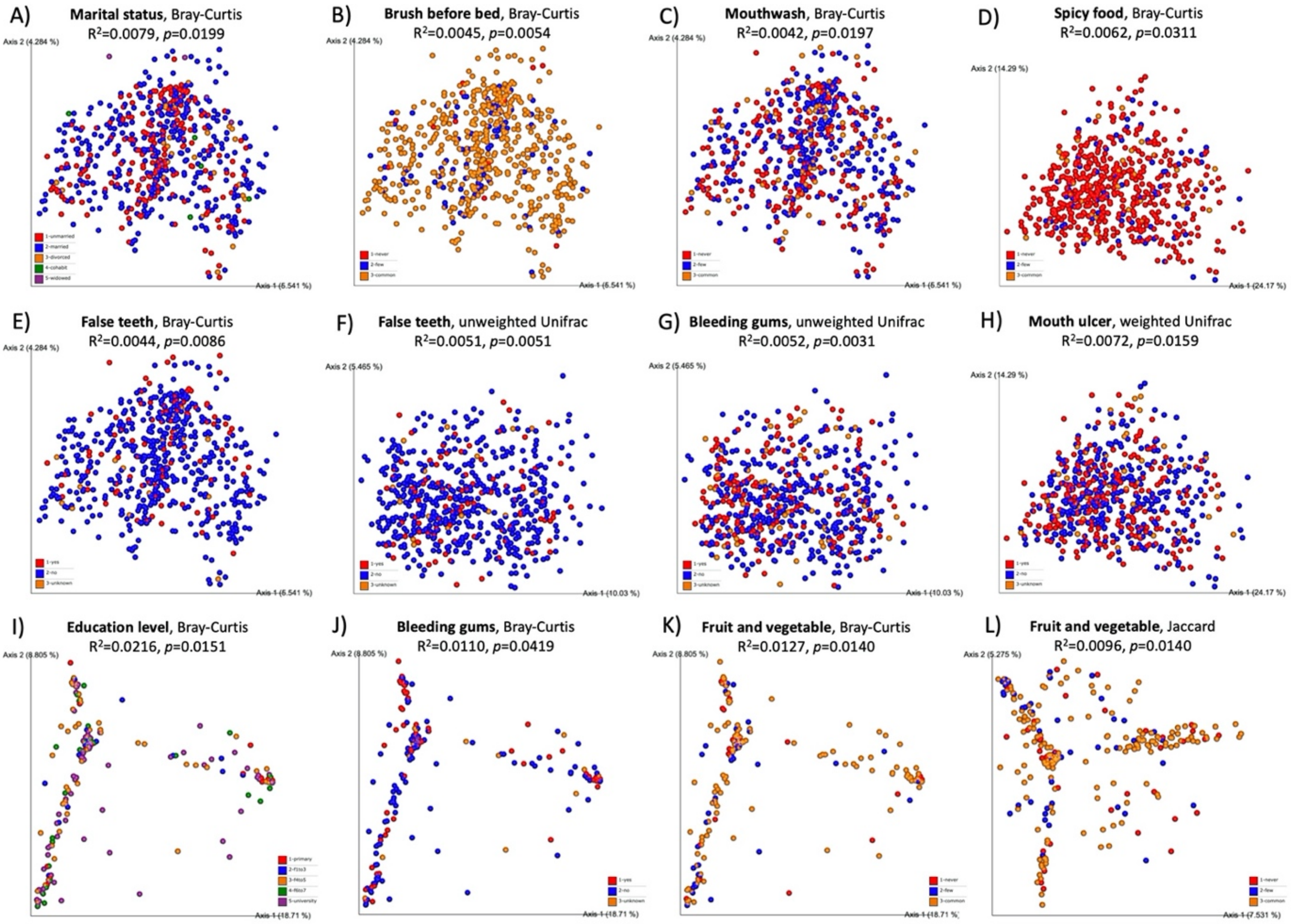

Figure S4. PCoA plots based on different distance metrics of the oral bacterial (A-H) and fungal (I-L) microbiome stratified by significant metadata variables after controlling for sex and age.

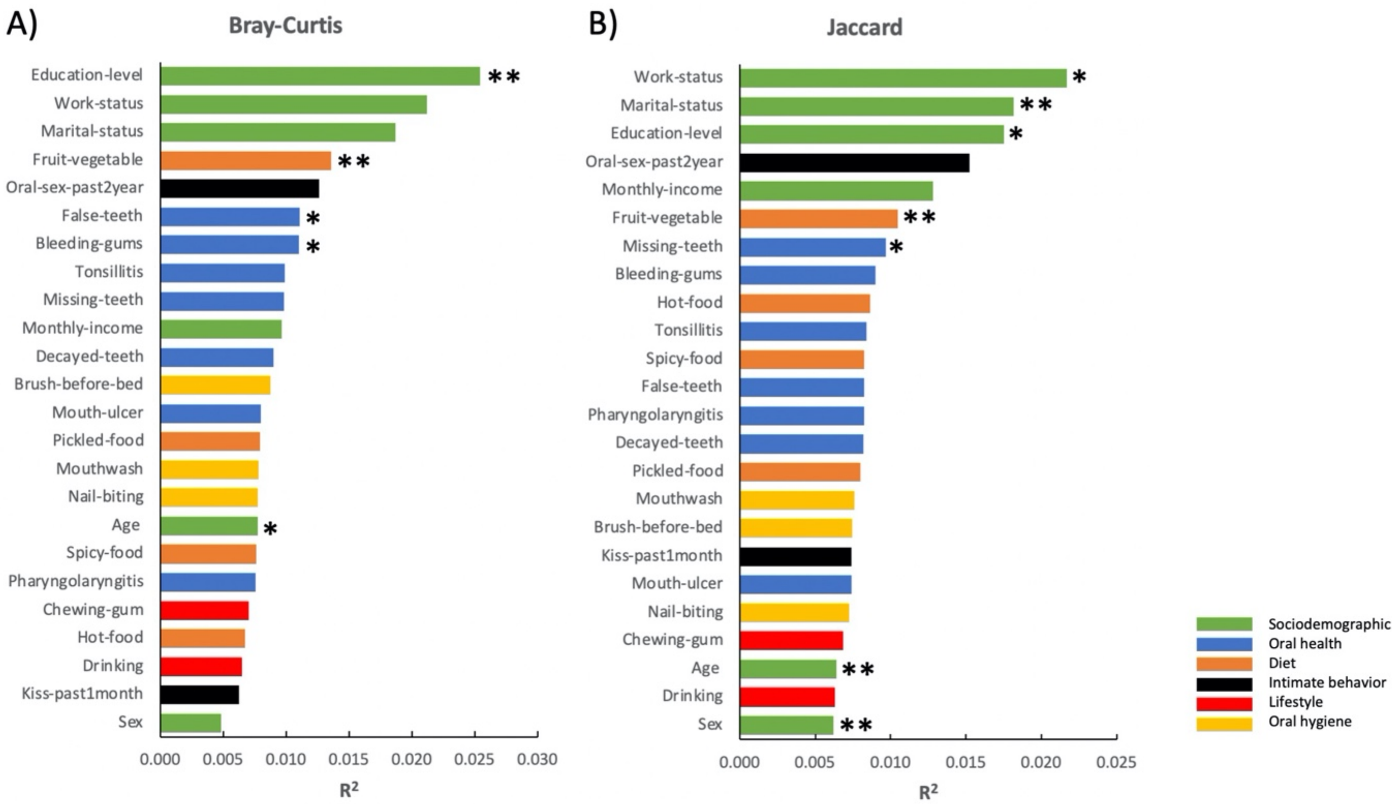

Figure S5. Effect size of metadata variables on the oral fungal microbiota composition based on Bray–Curtis dissimilarity (A) and Jaccard distance (B). Bars are coloured based on the category of the variables. *$p < 0.05$, **$p < 0.01$.

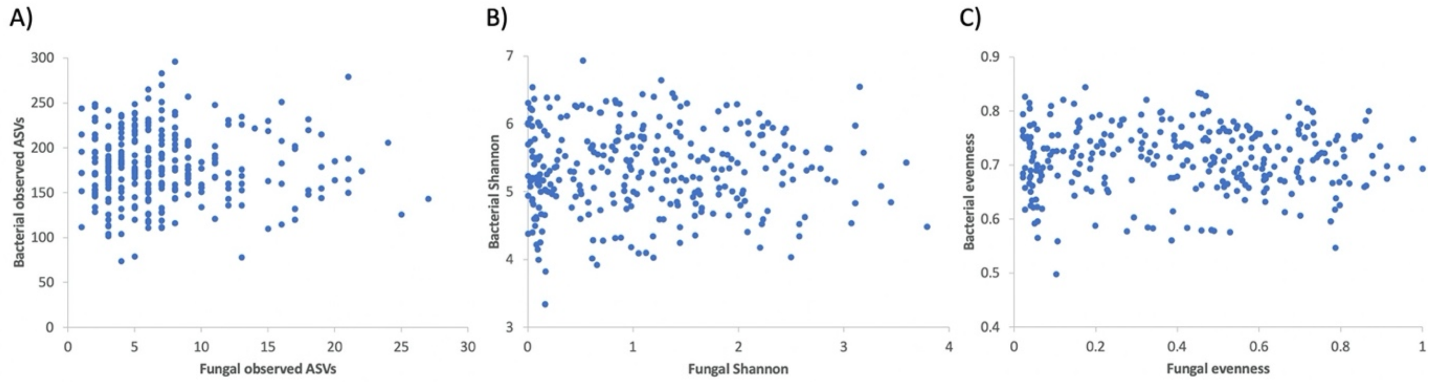

Figure S6. Spearman correlation analysis between alpha diversity of the oral bacterial and fungal microbiota based on number of observed ASVs (A), Shannon diversity (B), and Pielou's evenness (C).

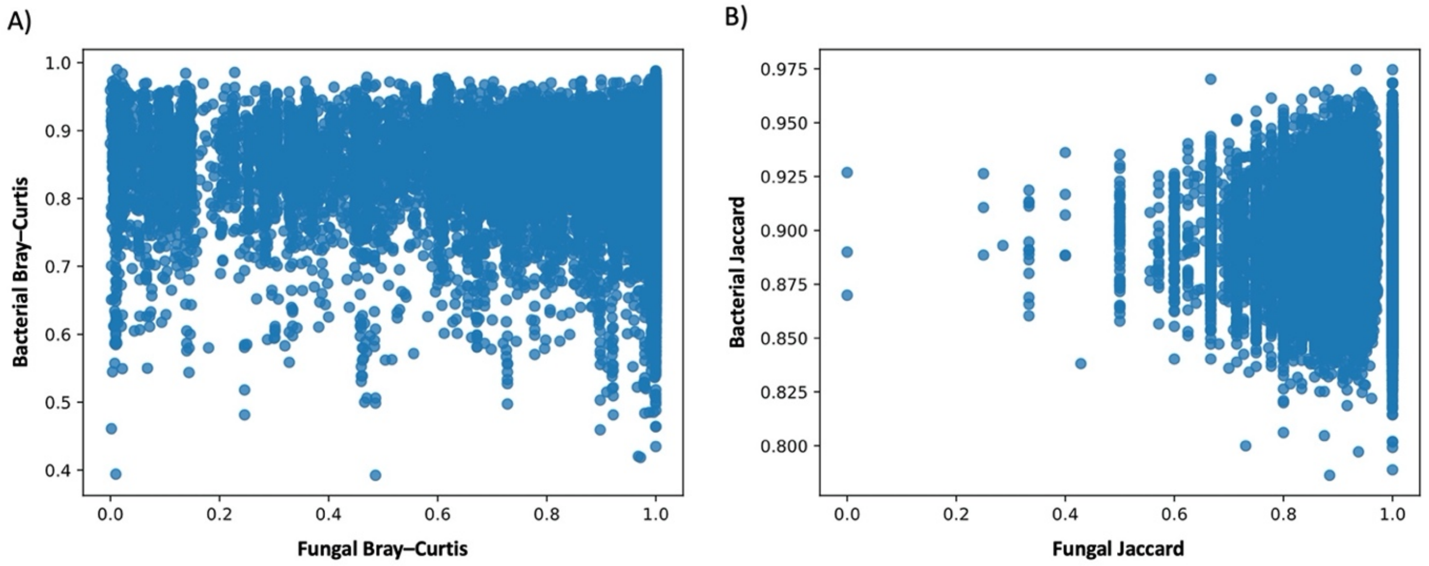

Figure S7. Spearman correlation analysis between beta diversity of the oral bacterial and fungal microbiota based on Bray–Curtis dissimilarity (A) and Jaccard distance (B).
